# Gut Microbiota and Probiotics/Synbiotics for Modulation of Immunity in Critically Ill Patients

**DOI:** 10.3390/nu13072439

**Published:** 2021-07-16

**Authors:** Kentaro Shimizu, Masahiro Ojima, Hiroshi Ogura

**Affiliations:** Department of Traumatology and Acute Critical Medicine, Osaka University Graduate School of Medicine, Osaka 565-0871, Japan; ojimarionet999@hp-emerg.med.osaka-u.ac.jp (M.O.); ogura@hp-emerg.med.osaka-u.ac.jp (H.O.)

**Keywords:** microbiota, gut, ICU, immune, ventilator, inflammation, probiotics, prebiotics, synbiotics, critically

## Abstract

Patients suffering from critical illness have host inflammatory responses against injuries, such as infection and trauma, that can lead to tissue damage, organ failure, and death. Modulation of host immune response as well as infection and damage control are detrimental factors in the management of systemic inflammation. The gut is the motor of multiple organ failure following injury, and it is recognized that gut dysfunction is one of the causative factors of disease progression. The gut microbiota has a role in maintaining host immunity, and disruption of the gut microbiota might induce an immunosuppressive condition in critically ill patients. Treatment with probiotics and synbiotics has been reported to attenuate systemic inflammation by maintaining gut microbiota and to reduce postoperative infectious complications and ventilator-associated pneumonia. The administration of prophylactic probiotics/synbiotics could be an important treatment option for preventing infectious complications and modulating immunity. Further basic and clinical research is needed to promote intestinal therapies for critically ill patients.

## 1. Introduction

**Figure 1:**
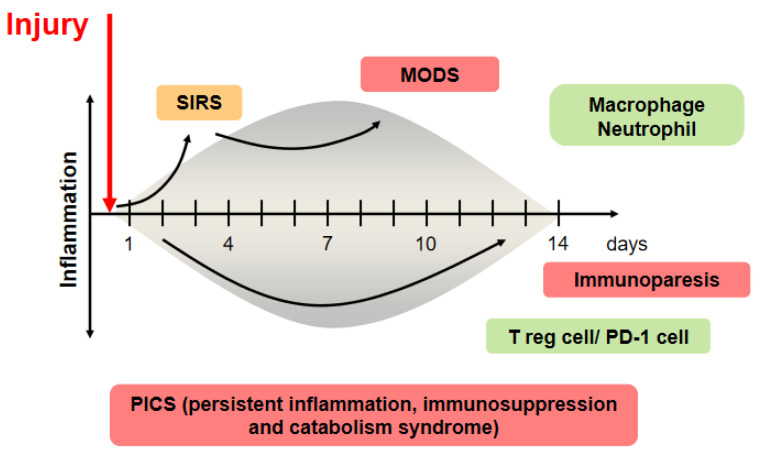
The concept of injury-induced imbalances of the immune system.

Patients suffering from critical illness can have a life-threatening host response to injuries, such as infection, trauma, burn, and cardiac arrest, that can lead to tissue damage, organ failure, and death. Sepsis management is a major challenge for healthcare systems throughout the world. About 50 million patients with sepsis were reported, with about 20% of all global deaths [1]. Trauma is the leading cause of death of children, adolescents, and younger adults [2]. These responses to injury affect cells and mediators of the innate and adaptive immune systems against pathogen-associated pattern molecules or damage-associated pattern molecules and have been referred to as systemic inflammatory response syndrome (SIRS) and compensatory anti-inflammatory response syndrome [3]. In contrast, prolonged SIRS suppresses Th1-type immune reactivity, and it also increases Th2-type cytokine production and activity of regulatory T cells. These conditions can easily progress to persistent inflammation, immunosuppression, and catabolism syndrome (PICS), which results in multiple immunologic and physiologic defects that are difficult to survive [4]. Modulation of host response against injury has been an important topic in this field (Figure 1).

Gut dysfunction, including the intestinal epithelium, intestinal immune system, and intestinal microbiota, is one of the causative factors of disease progression [5]. A loss of beneficial microbes, expansion of pathobionts, and loss of diversity are defined as “dysbiosis”, which can change host immunity [6]. Especially, the gut microbiota and microbial metabolites, such as short-chain fatty acids (SCFAs) and trimethylamine N-oxide, are associated with human diseases, such as allergic and immune disorders, cancer, cardiovascular disease, and neurological disorders [7]. This review summarizes how gut microbiota leads to systemic inflammation and intestinal therapy in the critically ill setting.

Once an insult occurs, it causes significant phenotypic changes in the immune system. The injured host develops inflammation following severe injury, which is called systemic inflammatory response syndrome (SIRS). If the SIRS state is prolonged, it progresses to the multiple organ dysfunction syndrome (MODS) stage. Inflammation and immunosuppression coincidentally begin at the onset of sepsis, and persistent inflammation, immunosuppression, and catabolism syndrome (PICS) develops in this concept [8]. This pro-inflammatory state has been shown to be mainly driven by macrophages, with regulatory T cells being the mediators of this compensatory response following injury.

## 2. Gut Origin of Sepsis and Systemic Inflammation

Injury to the gut has been known to affect systemic organs and the gut itself. The gut is the motor of multiple organ failure [9], and gut dysfunction is known as a causative factor in the progression of diseases such as sepsis, infection, shock, trauma, burn, and bleeding. In 1994, Moore et al. reported that in a rat intestinal reperfusion model, with 45-min occlusion of the supramesenteric artery following intraperitoneal injection of lipopolysaccharide, white blood cells accumulated and permeability in the lung increased, as did lung weight [10]. This result suggested that injury to the gut caused lung inflammation and systemic inflammation. One of the mechanisms is a bacterial translocation. In a mouse burn model, *Escherichia coli* was identified in the spleen and liver, five minutes after injury [11]. The mechanism has not been elucidated completely, but gut-derived factors were carried in the mesenteric lymph rather than the portal circulation by intestinal lymphatic division in a rat shock model [12]. In the intestinal epithelium, burn increases gut epithelial cell death by apoptosis and permeability [13]. The inhibition of apoptosis could attenuate survival in *P. aeruginosa* infection mouse models [14]. In addition, intestinal tight junction proteins, such as claudin-5 and occludin, decreased and could change gut permeability following injury in a cecal ligation puncture model [15]. These reports indicated that direct or indirect injury to the gut could cause intestinal damages and bacterial translocation, which lead to an inflammatory response in multiple organs.

In clinical research, bacterial translocation was present in the mesenteric lymph nodes of about 15% of patients with laparotomy [16] and of about 35% of patients with hepatectomy for biliary cancer [17]. Obligate anaerobes, such as *Clostridium coccoides* and *Bacteroides fragilis* groups, dominated in the lymph. These results suggest that the gut barrier following injury could allow bacteria and gut-derived mediators into the mesenteric lymph and into the bloodstream and activate a systemic inflammatory response.

## 3. Gut Microbiota in Critically Ill Patients

Under critically ill conditions, it is difficult to keep healthy gut microbiota because of disease and the various kinds of treatments, such as histamine H2 receptor blocker for bleeding prevention, catecholamines for blood pressure control, broad-spectrum antibiotics for infection control, and mechanical ventilation for respiratory failure. As shown in Table 1, the patients had about 10,000 times lower total anaerobes, including *Bifidobacterium* and *Lactobacillus*, and 100 times higher *Staphylococcus* as compared with those in healthy persons [18]. Total organic acids, acetic acid, and butyric acid derived from gut microbiota were significantly decreased compared with those in healthy persons (Table 2). Especially, butyric acid was almost depleted in the gut in critically ill conditions. Fecal pH was markedly increased in the patients compared with those in healthy persons. These results highlighted the deterioration of the gut microbiota and environment in the process of critical illness. The numbers of total obligate anaerobes and total facultative anaerobes were associated with bacteremia and mortality in patients with critically illness [19].

**Figure 2:**
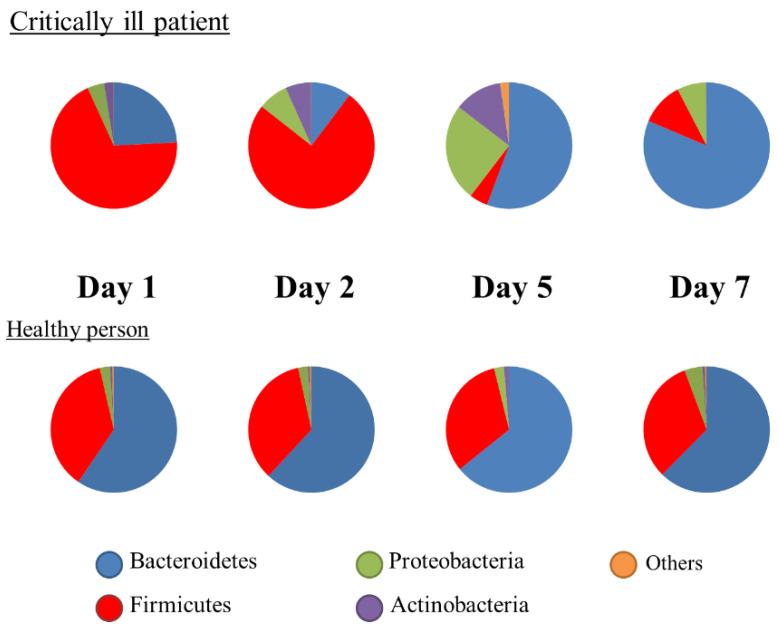
Changes in the composition of gut microbiota. Representative cases showing the proportions of microbiota.

**Figure 3:**
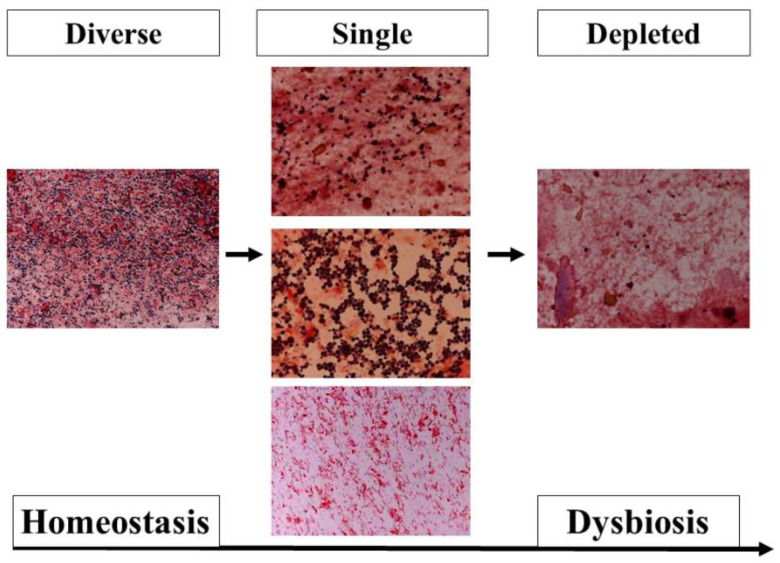
Pattern classification of fecal Gram staining. (**Left**) In the diverse pattern, many kinds of bacteria cover the field. (**Middle**) In the single pattern, a few kinds of bacteria dominantly cover the field. These images show that Gram-positive cocci (**Upper**), Gram-negative rods (**Middle**), and fungus (**Bottom**) dominantly cover the field. (**Right**) In the depleted pattern, most bacteria are depleted in the field. The dysbiosis progresses from a diverse pattern to a single pattern, and from a single pattern to a depleted pattern.

Metagenomic analysis using the 16S rRNA gene has been developed and shown to be different with ethnicity, diet, nutrition, lifestyle, and disease [20]. This method is not a quantitative analysis; rather, the results were expressed as proportions. At the phylum level, the Firmicutes or Bacteroidetes were predominant in healthy adults. In intensive care unit (ICU) patients, both phyla were altered significantly (Figure 2), and the ratio of Bacteroidetes to Firmicutes (B/F ratio) was associated with mortality [21]. The gut microbiota begins to change within the first 6 h [22]. Ojima et al. reported that gut microbiota under broad-spectrum antibiotics dramatically changed and stabilized within the first week [23]. To assess biomarkers at the bedside, metagenomic analysis has not been adequately used until now. However, fecal gram staining can be used as a quick marker for dysbiosis [24] (Figure 3).

## 4. Immune Reactions through Gut Microbiota

The human gut microbiota has no less than 100 times more bacterial than human genes; therefore, metabolism is a combination of microbial- and human-derived actions [25]. In this mutualistic relationship, there is immunological tolerance of many bacteria. Obligate anaerobic bacteria, a main gut microbiota, are the main inhibitors of bacterial overgrowth, which is defined as ‘‘colonization resistance” [26]. In addition, the gut microbiota has a role in producing immune signals that affect the host’s metabolism, immunity, and response to infection by the immune system [27]. The germ-free mouse has a smaller mucus barrier, Peyer’s patches, and lamina propria and also decreased T cells and B cells compared with the normal mouse [28]. The signals from microbial components and metabolites, such as SCFAs, coupled with pathogen recognition receptors, such as Toll-like receptors and nucleotide oligomerization domain (NOD)-like receptors (NLRs) in the intestine, orchestrate host-microbiota interaction and multifactorial diseases. In the absence of the microbiota, reduced myeloid-cell development in the bone marrow causes delayed clearance of systemic bacterial infection. For adaptive immunity, Ivanov et al. revealed that segmented filamentous bacteria induce Th17 cells [29]. Atarashi et al. revealed that 17 species of bacteria, including *Clostridiales,* induced regulatory T cells [30]. ICU patients were significantly lower in the proportions of the class *Clostridia* than the healthy controls [31]. IFN-γ-producing CD8 + cells were induced by 11 species of bacteria included in the phyla Bacteroidetes, Firmicutes, and Fusobacterium [31]. Thus, the gut microbiota can help to shape the balance of immune cells and modulate immune status [32]. In autopsy cases of sepsis, the numbers of CD4 + T cells, CD8 + T cells, and HLA-DR+ cells were decreased, whereas those of PD-1, regulatory T cells, and myeloid-derived suppressor cells were increased [33]. As the extreme balance of Bacteroidetes and Firmicutes ratio was associated with mortality, the disrupted balance of commensal gut microbiota following injury might be associated with imbalance of immunity. There is a missing link between gut microbiota and systemic immunity, but residual pathogenic bacteria might induce an inflammatory reaction, or a depleted microbiota might decrease an innate immune response, resulting in prolonged immunosuppression (Figure 1).

## 5. Effects of Probiotics and Synbiotics during Critical Illness

### 5.1. Influence of Probiotics on Gut and Immunity

Probiotics are defined as live microorganisms, which confer a health benefit on the host [34]. Probiotics, most commonly *Lactobacillus* and *Bifidobacterium*, have been shown to have preventive effects in many kinds of diseases, including antibiotic-induced diarrhea, acute diarrhea, and necrotizing enterocolitis [35]. Prebiotics are currently defined as non-digestible food ingredients that beneficially affect the host by selectively stimulating the growth and/or activity of one or a limited number of bacteria in the colon [36]. Synbiotics are combinations of probiotics and prebiotics. For critically ill patients, synbiotics have prophylactic effects for complications of abdominal surgery, trauma, and ventilator-associated pneumonia (VAP) [37].

The mechanisms of probiotics have been explained with improving barrier function and immunity through the actions of cell components and metabolites of probiotics [38,39]. There are direct effects, including microorganism-associated molecular patterns (MAMPs) and pattern recognition receptors (PRRs), in the gut mucosa [40]. MAMPs involve flagellin, lipopolysaccharide, lipoteichoic acid, peptidoglycan, etc. For example, flagellins of the probiotic *E. coli* Nissle 1917 were shown to induce beta-defensin via Toll-like receptor 5 [41]. Peptidoglycan from the gut translocates to the circulation and increases the killing capacity of neutrophils via NOD1 [42]. For probiotic metabolites, probiotic bifidobacteria were shown to produce a high concentration of acetic acid and to lower the pH of the gut in a enterohemorrhagic *E. coli* 0157 mouse model [43]. *Bifidobacterium breve* have a positive correlation with acetic acid levels and intestinal epithelium expression of tight junction-related genes [44]. *L. casei* increased pulmonary natural killer cell activity, and interleukin-12 production was increased in a mouse model of influenza virus infection [45]. In a mouse model of diarrhea associated with clindamycin antibiotics administration, *C. butyricum* decreased the level of inflammatory cytokines and intestinal barrier-related proteins, such as IL-6, IFN-γ, mucin-2 in the colon [46]. *Lactobacillus* increased regulatory T cells with better survival in a mouse model of pseudomonas pneumonia [47]. These data suggest that probiotics might modulate the host response and prevent systemic inflammation.

### 5.2. Effectiveness of Probiotics and Synbiotics for Diarrhea

Diarrhea has been reported to prolong ICU stay and increase mortality in the ICU [48]. Hickson et al. reported that in 135 hospital patients with antibiotics and *L.* *casei*, *L. bulgaricus*, and *Streptococcus thermophiles*, 12% in the probiotic group exhibited diarrhea compared with 34% in the control group [49]. A meta-analysis revealed that *Saccharomyces boulardii*, *L. rhamnosus* GG, or *B. longum* were used to show the prevention of diarrhea in the hospital setting [50].

In ICU settings, Bleichner et al. reported that in 128 ICU patients, the number of days with diarrhea was reduced in the *S. boulardii-*treated group [51]. Shimizu et al. reported that *B. breve*, *L. casei*, and galactooligosaccharides had less complications of diarrhea and ventilator-associated diarrhea in 178 ventilated critically ill patients [52]. Prophylactic probiotics and synbiotics might keep gut microbiota and reduce the incidence of diarrhea. For refractory diarrhea, fecal microbiota transplantation (FMT) is a strong reconstruction of the gut microbiota [53], which has been recommended for patients with multiple recurrences of *C. difficile* infection [54]. A case of antibiotics-associated diarrhea was also reported among patients with diarrhea of more than 5 L/day, in which the gut microbiota was recovered and symptoms were resolved by FMT [55]. In severe gut microbiota disruption, more effective probiotics/synbiotics could be required in the ICU.

### 5.3. Prophylactic Effect of Probiotics and Synbiotics on Critical Illness

The effects of probiotics/synbiotics are based upon the alteration of gut microbiota and environment in critically ill patients. Those patients who received probiotics had significantly greater levels of *Bifidobacterium*, *Lactobacillus*, and microbial products, particularly SCFAs, than those who did not receive probiotics [56]. In meta-analyses, probiotics have been reported to reduce infectious complications of surgical procedures [57] and trauma [58]. Although the cause of upregulated immunity has not been elucidated, Sugawara et al. reported that patients taking probiotics before hepatectomy had more natural killer cell activity, increased lymphocyte counts, and lower IL-6 levels after hepatectomy than those not taking probiotics [59].

**Figure 4:**
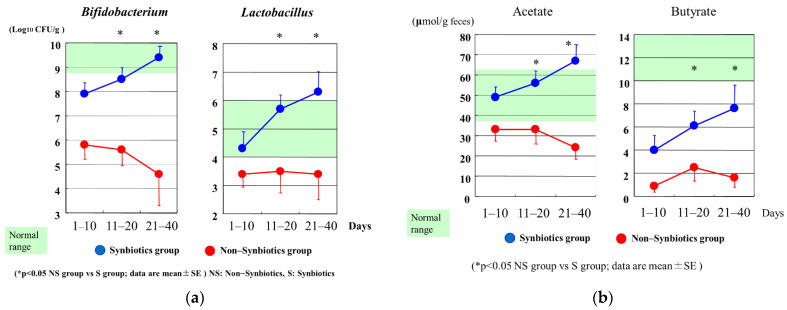
(**a**) Serial changes in *Bifidobacterium* and *Lactobacillus* after admission. *Bifidobacterium* and *Lactobacillus* counts in the Synbiotics group were significantly increased compared with those in the Non–Synbiotics group. (**b**) Serial changes in acetate and butyrate after admission. Acetate and butyrate in the Synbiotics group were significantly increased compared with those in the Non–Synbiotics group.

**Figure 5:**
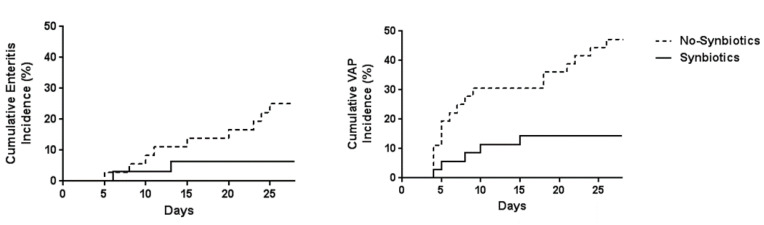
Effects of synbiotics on infectious complications. The cumulative incidence of enteritis and ventilator-associated pneumonia (VAP) were significantly lower in the Synbiotics group than in the Non-Synbiotics group by log-rank test (*p* < 0.05). (Cited from [62]).

**Figure 6:**
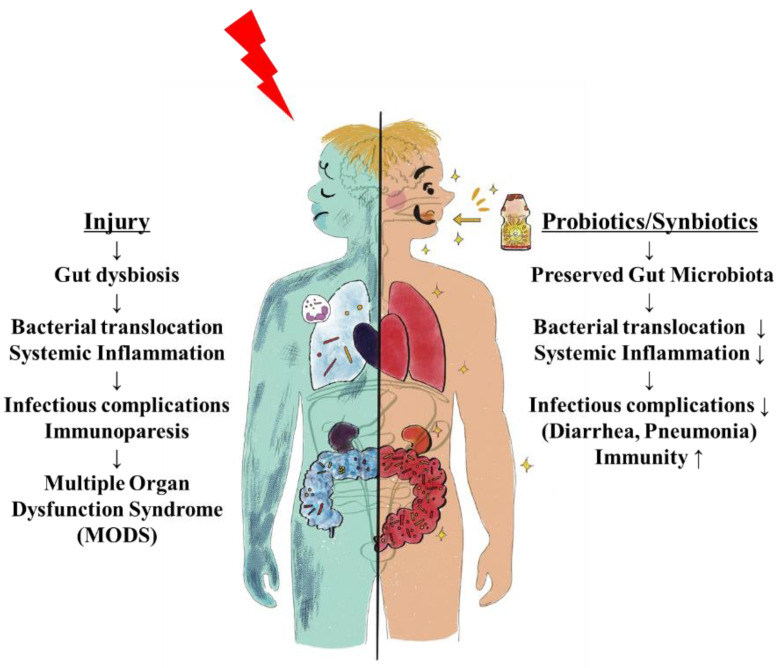
Gut origin hypothesis and Probiotics/Synbiotics. Deteriorated microbiota following injury cause systemic inflammation and infectious complications including pneumonia. If inflammation continues, immunoparesis and multiple organ failures develop. Probiotics and synbiotics help to maintain gut microbiota and prevent infectious complications and their subsequent sequelae.

In the ICU setting, analysis of fecal flora confirmed that critically ill patients in the synbiotics group had significantly greater levels of beneficial *Bifidobacterium*, *Lactobacillus*, and total organic acids, particularly SCFAs, than those in the non-synbiotics group [60] (Figure 4). Synbiotics maintain the gut flora and environment and decrease the incidence of diarrhea and VAP in ventilated patients with sepsis [61] (Figure 5). In a meta-analysis, Batra et al. reported that in ventilated critically ill ICU patients, the administration of probiotics reduced their incidence of VAP, the duration of mechanical ventilation, length of ICU stay, and in-hospital mortality [62]. This research indicated that the administration of probiotics and synbiotics could maintain gut microbiota and upregulate immunity (Figure 6).

The new coronavirus disease (COVID-19) causes gastrointestinal symptoms as well as respiratory symptoms. The severe acute respiratory syndrome coronavirus 2 (SARS-CoV-2) uses angiotensin-converting enzyme 2 (ACE2). A deficiency in murine ACE2, which encodes a key regulatory enzyme of the renin–angiotensin system, results in highly increased susceptibility to diarrhea and intestinal inflammation [63]. In gut microbiota of COVID-19 patients, the decrease in normal gut microbiota bacteria, such as *Eubacterium ventricosum*, *Faecalibacterium prausnitzii*, *Reseburia*, and *Lachnospiraceae,* and the increase of opportunistic bacteria, such as *C. hathewayi*, *Actinomyces viscosus*, and *B. nordii*, was observed [64]. Probiotics/synbiotics are one of the promising therapies for COVID-19 to maintain gut microbiota and prevent the exacerbation of pneumonia [39]. In 200 adults with severe COVID-19 pneumonia, patients treated with several kinds of probiotics were associated with a reduced risk for death [65]. Intestinal therapy for COVID-19 might be important to prevent pneumonia and systemic inflammation.

### 5.4. Gut Dysmotility and Limitations for Intestinal Therapy

Gut motility in critically ill patients is often suppressed by various factors, for example, ischemia, analgesics, adrenergic drugs, fluid therapy, and diseases such as diabetes [66,67]. This intestinal motility failure leads to increased gut permeability for mediators and bacteria and the development of SIRS. Gastrointestinal complications in critically ill patients occurred about 60%, and enteral nutrition was stopped about 15% [68]. ICU patients with feeding intolerance had significantly deteriorated gut microbiota compared to those patients without feeding intolerance (*p* < 0.05) [69]. Patients with feeding intolerance had significantly higher rates of bacteremia and mortality. Interstitial cells of Cajal, which has a role of gut motility, form an extensive network associated with the myenteric plexus in the enteric nervous system [70]. These cells in critically ill patients were almost decreased in the colon compared with those in the controls [71].

Pancreatitis is one of the critical illnesses in the abdomen, and gastrointestinal dysmotility can cause feeding intolerance and bacterial translocation [72]. Olah et al. [73] reported that the incidence of infectious complications with *Lactobacillus plantarum* decreased more than those without *L. plantarum* (4.5% vs. 30.4%). In contrast, Besselink et al. [74] reported that bowel ischemia and mortality rates in patients with six kinds of bacteria were significantly higher than those in patients without these bacteria (16 vs. 6%). However, in this study, the incidence of infectious complications showed no significant difference, and no bacteremia was caused from the administered bacteria. In addition, this study has been criticized from multiple perspectives [75,76]. Extensive burn can cause SIRS, sepsis, and multiple organ dysfunction syndrome. In a case of non-obstructive ileus in burn, the numbers of gut microbiota, mainly those of *Bifidobacterium*, decreased and *Pseudomonas* and *Candida* increased in major burn patients [77]. These results indicate that critically ill patients with gastrointestinal dysmotility have altered gut microbiota that could lead to an ‘‘undrained abscess’’. These studies further suggest that the effect and safety of probiotics differ with the bacteria administered and intestinal tolerance. Probiotics and synbiotics might not be indicated for severe intestinal dysmotility. Further studies are needed to determine an appropriate therapeutic indication for severe abdominal inflammation.

## 6. Summary

The gut microbiota has a role in maintaining host immunity. Deterioration of the gut microbiota in critical illnesses can lead to systemic inflammation response syndrome and multiple organ dysfunction syndrome.

Probiotics/synbiotics treatment can maintain the disrupted gut microbiota and reduce infectious complications in critically ill patients. Further basic and clinical research will be required to promote intestinal therapies for critically ill patients.

## Figures and Tables

**Table 1:** Fecal flora in patients with severe SIRS.

	SIRS Patients	Normal
Total obligate anaerobes	8.3 ± 2.3 *	10.5 ± 0.5
*Bacteroidaceae*	7.3 ± 3.0 *	10.1 ± 0.4
*Bifidobacterium*	4.8 ± 3.3 *	9.6 ± 0.7
*Clostridium*	2.1 ± 1.0	2.1 ± 0.7
*Veillonella*	3.1 ± 1.8 *	7.0 ± 1.2
Total facultative anaerobes	7.8 ± 1.4	7.5 ± 0.4
*Lactobacillus*	2.7 ± 1.5 *	5.0 ± 1.0
*Enterobacteriaceae*	4.1 ± 2.7 *	7.4 ± 0.8
*Enterococcus*	6.4 ± 2.5	7.0 ± 0.9
*Staphylococcus*	5.3 ± 1.7 *	2.7 ± 0.8
*Pseudomonas*	2.8 ± 1.4 *	ND
*Candida*	2.5 ± 1.0	2.0 ± 0.5

(* *p* < 0.05 NS group vs. S group; data are mean ± SE) NS: Non–Synbiotics, S: Synbiotics.

**Table 2:** Fecal organic acid concentrations and pH in patients with severe SIRS.

	SIRS Patients	Normal
Total organic acid	30.3 ± 20.3 *	88.4 ± 21.2
*Succinic acid*	2.0 ± 2.5	0.9 ± 1.2
*Lactic acid*	3.8 ± 5.5	0.5 ± 0.3
*Formic acid*	1.7 ± 2.9	0.4 ± 0.3
*Acetic acid*	18.7 ± 15.9 *	50.8 ± 13.1
*Propionic acid*	2.5 ± 4.6 *	18.7 ± 6.8
*Isobutyric acid*	0.1 ± 0.5	1.1 ± 0.3
*Butyric acid*	0.9 ± 2.3 *	16.6 ± 6.7
*Isovaleric acid*	0.5 ± 1.9	1.4 ± 0.7
*Valeric acid*	0.1 ± 0.7	0.6 ± 0.4
pH	7.4 ± 0.6 *	6.6 ± 0.3

(* *p* < 0.05 NS group vs. S group; data are mean ± SE).

## Data Availability

Not applicable.
